# Meningitis-Retention Syndrome After Traumatic Subarachnoid Hemorrhage: A Case Report

**DOI:** 10.7759/cureus.93965

**Published:** 2025-10-06

**Authors:** Kiyohiro Oshima, Masato Murata, Yusuke Sawada, Yumi Ichikawa, Keiko Kawai-Kowase

**Affiliations:** 1 Department of Emergency Medicine, Gunma University Graduate School of Medicine, Maebashi, JPN; 2 Department of Emergency and Critical Care, National Hospital Organization Takasaki General Medical Center, Takasaki, JPN; 3 Department of General Medicine, Gunma University Graduate School of Medicine, Maebashi, JPN

**Keywords:** aseptic meningitis, fever, traumatic subarachnoid hemorrhage, uresiesthesia, urinary retention (ur)

## Abstract

A 59-year-old male, who had been hospitalized for 13 days due to a traumatic subarachnoid hemorrhage, presented with a fever > 38ºC two days after discharge. He also experienced urinary retention five days after discharge. A lumbar puncture was performed on the sixth day after discharge because his Kernig’s sign became slightly positive. Cerebrospinal fluid analysis showed lymphocytic pleocytosis, while the etiological examination was negative. Finally, a diagnosis of meningitis-retention syndrome (MRS) was made. He was admitted to our department and received antiviral treatment for 14 days. He was discharged on the 23rd day, and the urinary catheter was removed about one month later. The patient’s clinical course has remained uneventful 62 months after discharge.

MRS is a para-infectious disease characterized by the combination of aseptic meningitis, cerebrospinal fluid pleocytosis, and acute urinary retention with no other neurological abnormalities. The actual prevalence of MRS is likely underestimated, making early diagnosis challenging. MRS should be considered in the differential diagnosis of patients presenting with fever and urinary retention.

## Introduction

Meningitis-retention syndrome (MRS) is a para-infectious disease characterized by the combination of aseptic meningitis and acute urinary retention with no other abnormalities [1, 2]. MRS cases generally present without encephalitic symptoms or signs, which distinguishes MRS from acute disseminated encephalomyelitis (ADEM) [3]. Another distinguishing feature is that MRI of the brain and spinal cord reveals no abnormalities in MRS [3].

Although several MRS cases have been reported in the literature, its actual prevalence is underestimated [3]. We herein report a case diagnosed as MRS following a traumatic brain injury. Patient consent was obtained for the use of their data in this case report.

## Case presentation

**Figure 1:**
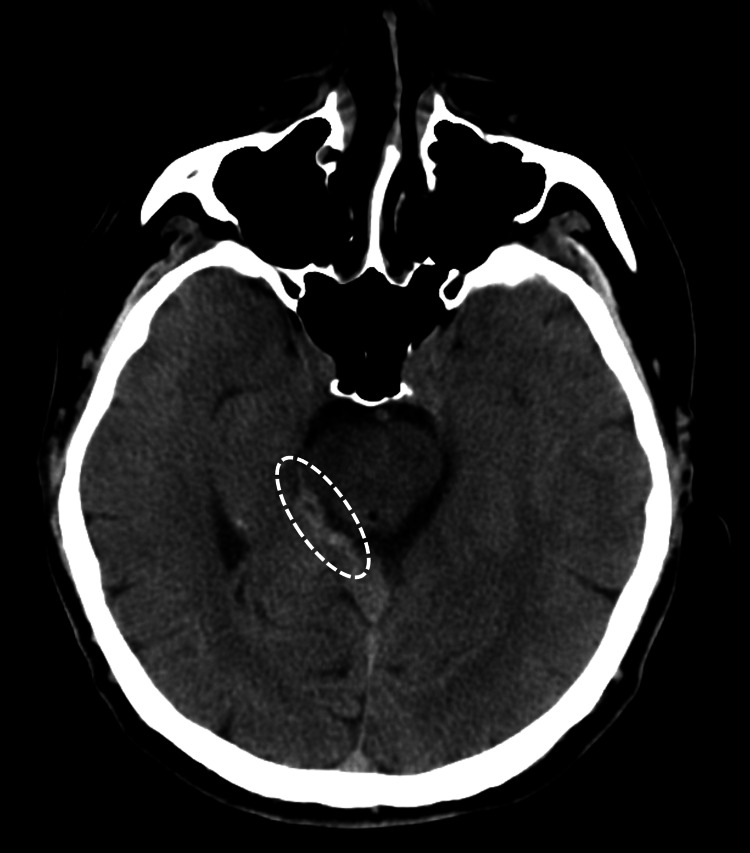
Brain CT of traumatic subarachnoid hemorrhage. Hematoma located in the right ambient cistern, measuring approximately 30 mm × 3 mm (white oval with broken line).

A 59-year-old male, who had undergone a right radical orchiectomy for testicular cancer at age 38 without any other important medical history, was admitted to the Neurosurgery Department of our hospital for 13 days due to a traumatic subarachnoid hemorrhage (tSAH, hematoma located in the right ambient cistern, approximately 30 mm × 3 mm in size; Grade 1 in the Morris-Marshall classification) caused by a traffic accident (Figure 1).

His clinical recovery was uneventful, and he was discharged without any consciousness disorder or paralysis. There were no clinical symptoms or imaging findings compatible with CSF fistula at discharge. Two days later, he presented to our ER with a fever > 38ºC. He was initially thought to have a common cold because there were no abnormalities in his physical examination except for fever, and his blood test results were unremarkable. On the 3rd day after discharge, he consulted a different doctor because of continued fever, headache, and vomiting, and was subsequently transferred to the General Medicine Department of our hospital for further investigation and treatment on the 4th day after discharge. His physical findings were as follows: height, 177.4 cm; body weight, 60.4 kg; GCS, E4V5M6; body temperature, 38.6ºC; pulse, 117/min, regular; blood pressure (BP), 168/94 mmHg. He complained of headache but had a negative Kernig’s sign, no neck stiffness, and no other neurological abnormalities. Blood tests showed no elevation of inflammatory parameters (WBCs, 6,600/μL; CRP, 0.03 mg/dL). Chest radiograph and brain CT scans were normal. A neurosurgeon also examined the patient and judged the possibility of meningitis to be low. He returned home the same day but came back to our hospital the following day because he began experiencing urinary disturbance with micturition desire overnight. At that time, his physical findings were: alert; body temperature, 39.6ºC; BP, 135/87 mmHg; SpO₂, 98% (ambient air). His headache had improved, and he showed no signs of meningeal irritation, as there was no neck stiffness or positive Kernig’s sign. Urinary tests revealed no evidence of urinary infection. The urologist examined the patient, and ultrasound demonstrated a large volume of residual urine in the bladder in the absence of prostatitis, epididymitis, or bilateral hydronephrosis. Urinary retention was diagnosed, and about 1,000 mL of residual urine was extracted via urinary catheter. On the 6th day after discharge, a lumbar puncture was performed because Kernig’s sign became slightly positive, and CSF examination revealed lymphocytic pleocytosis and hyperproteinorrachia without cytologic evidence of malignancy (Table 1).

**Table 1:** Examination of blood and cerebrospinal fluid. Ht: Hematocrit; Hb: Hemoglobin; Plt: Platelets; TP: Total protein; Alb: Albumin; T-Bil: Total bilirubin; AST: Aspartate aminotransferase; ALT: Alanine aminotransferase; LDH: Lactate dehydrogenase; Amy: Amylase; BUN: Blood urea nitrogen; Cr: Creatinine; Na: Sodium; K: Potassium; Cl: Chloride; BS: Blood sugar; CRP: C-reactive protein; PCT: Procalcitonin.

Item	Value	Unit	Item	Value	Unit
Ht	40.5	%	Na	139	mEq/L
Hb	14.1	g/dL	K	3.7	mEq/L
RBC	4.64 × 10⁶	/μL	Cl	100	mEq/L
WBC	6,200	/μL	BS	109	mg/dL
Plt	195 × 10³	/μL	CRP	0.08	mg/dL
TP	6.8	g/dL	PCT	<0.02	ng/mL
Alb	3.8	g/dL	Findings of cerebrospinal fluid		
T-Bil	1.2	mg/dL	Color	None	
AST	24	U/L	Cell count	25	/μL
ALT	20	U/L	Protein	122	mg/dL
LDH	179	U/L	Sugar	40	mg/dL
Amy	92	U/L	Monocytes / Polynuclear cells	95 / 5	%
BUN	15	mg/dL	Initial pressure	115	mmH₂O
Cr	0.68	mg/dL	Final pressure	110	mmH₂O

**Figure 2:**
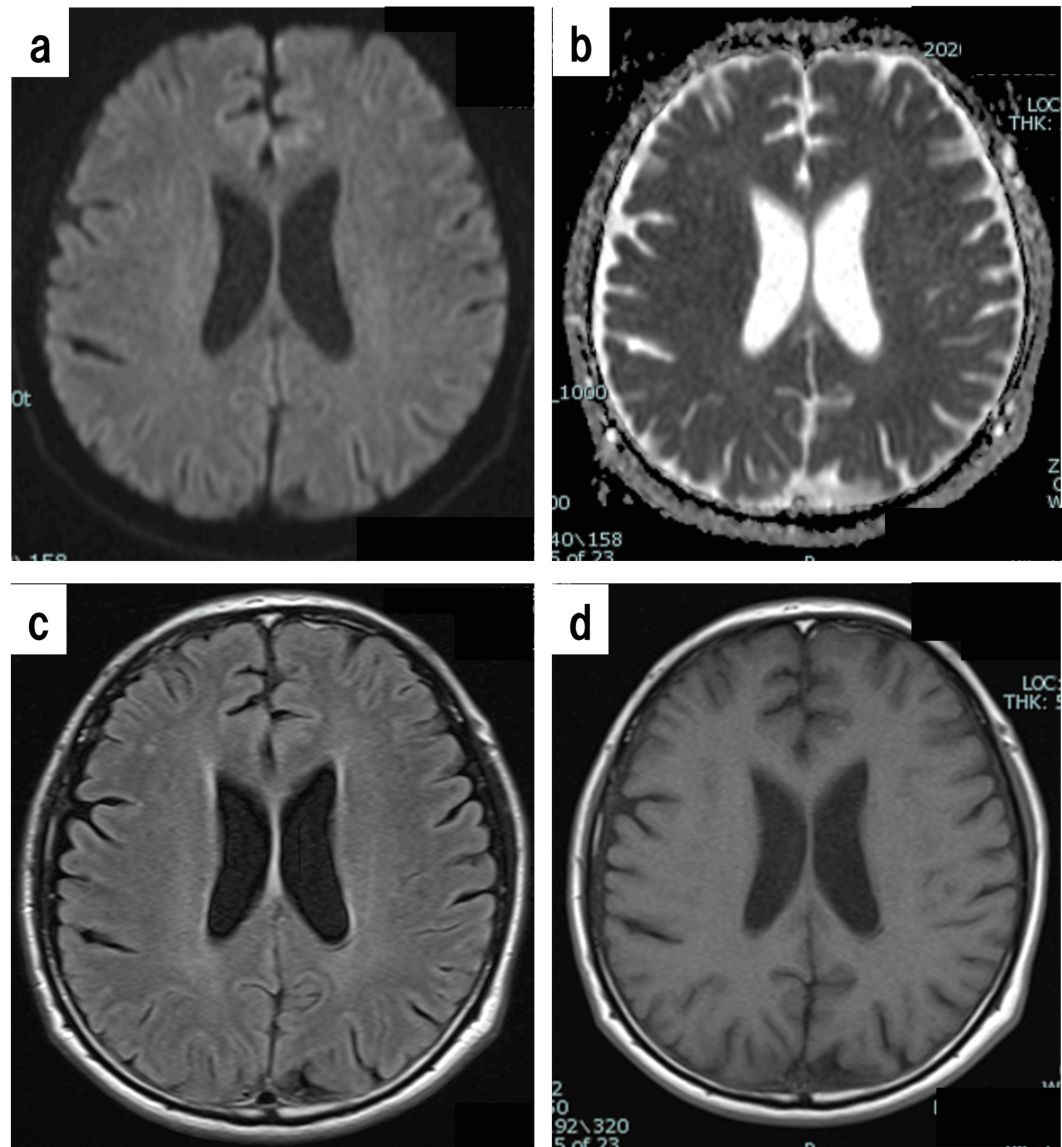
Brain MRI performed on the 6th day after discharge. a: Axial diffusion-weighted image; b: Apparent diffusion coefficient (ADC) image; c: Fluid-attenuated inversion recovery (FLAIR) image; d: T1-weighted (T1WI) image. No abnormalities were found on the brain MRI scans.

Blood test results from the same day (2.5 hours before lumbar puncture) are also shown in Table 1. Brain MRI scans performed on that day showed no abnormalities (Figure 2).

The diagnosis of aseptic meningitis with secondary urinary retention, otherwise known as MRS, was made, and the patient was admitted to our department.

**Figure 3:**
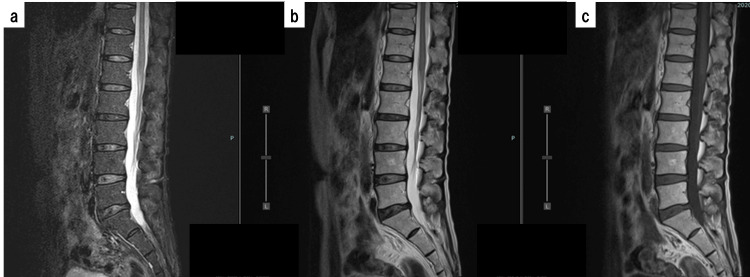
Lumbar spine MRI performed on the 5th day of hospitalization. a: Sagittal short τ inversion recovery (STIR) image; b: T2-weighted (T2WI) image; c: T1-weighted (T1WI) image. No abnormalities were found on the lumbar spine MRI scans.

On the 1st day of hospitalization, the patient started receiving IV acyclovir (10 mg/kg/8 hr) since HSV is one of the most common causes of aseptic meningitis in MRS [4]. However, both polymerase chain reaction and IgG antibody for HSV in CSF were negative, and culture tests, including fungal and mycobacterial cultures, were also negative. Thus, antiviral therapy was terminated after 14 days. A lumbar spine MRI performed on the 5th day of hospitalization showed no abnormalities, including no evidence of malignancy (Figure 3).

The patient’s fever began to decrease after the 10th day of hospitalization, and he was discharged from our department on the 23rd day of hospitalization, as he no longer had fever or headache. He continued to experience urinary retention after his fever dissipated; therefore, he was discharged with a urinary catheter, which was removed about one month after discharge. The patient’s clinical course has remained uneventful 62 months after discharge from our department.

## Discussion

MRS was originally described in 2005 by Sakakibara R et al., who defined it as a peculiar disorder characterized by aseptic meningitis, typically without any clear causative agent, and associated with acute urinary retention [1]. Meanwhile, Mertz G and Corey L had already reported in 1984 that primary herpes infections in adults could include complications such as aseptic meningitis and urinary retention [5]. Hiraga A and Kuwabara S suggested that MRS occurred in 8% of patients with aseptic meningitis [2]. However, Pellegrino F et al. documented the existence of only 29 MRS cases, including their own, after a literature review of MEDLINE, EMBASE, PubMed, Orphanet, and Cochrane Library databases in 2023 [3]. We also reviewed PubMed for MRS case reports and found only 30 cases (search performed on June 27, 2025). The actual prevalence of MRS is still underestimated, making early diagnosis of this syndrome difficult [3].

Mertz G and Corey L who reported the combination of aseptic meningitis and urinary retention in 1984, proposed that urinary retention could be explained by sacral radiculopathy or autonomic dysfunction [5]. To date, the underlying mechanism of urinary retention in MRS has not been completely elucidated, but possible explanations include spinal shock due to inflammation of the upper motor neurons of the spinal cord, meningeal irritation, direct viral or bacterial entry, and the onset of acute disseminated encephalomyelitis after viral or bacterial infection [4, 6]. Tateno F et al. conducted a urodynamic study in a patient with MRS and found that the initially areflexic detrusor became overactive, suggesting upper motor neuron dysfunction of the bladder [7]. Retention usually improves within 1-2 weeks without specific treatment [2]. In the present case, urinary retention also improved naturally over approximately 50 days.

The relationship between tSAH and aseptic meningitis in our patient remains unclear. In fact, tSAH is not generally cited as a cause of meningitis, including aseptic meningitis [8]. In this case, aseptic meningitis occurred at least 13 days after the tSAH; therefore, the occurrence of aseptic meningitis may not be associated with tSAH.

Signs of meningeal irritation such as neck stiffness, Brudzinski's sign, photophobia, and phonophobia were absent, except for a slightly positive Kernig’s sign. In addition, it was difficult to diagnose meningitis based solely on cerebrospinal fluid findings in the context of post-tSAH status. However, we speculated that MRS was present based on the initial symptoms of fever and urinary retention, and treatment was initiated accordingly.

## Conclusions

We reported a case of MRS after tSAH. MRS is rare and is often misdiagnosed as a UTI. Its actual prevalence is still underestimated, making early diagnosis difficult. Although MRS generally follows a benign and self-remitting course, management of acute urinary retention is essential. Therefore, quick and accurate diagnosis of MRS is required, and it should be included in the differential diagnosis of patients presenting with fever and urinary retention. Further studies are needed to clarify the pathophysiology of MRS.
